# Effect of intravenous immunoglobulin combined with azithromycin sequential therapy on clinical outcomes and immune response in children with *Mycoplasma pneumoniae* pneumonia: a retrospective study

**DOI:** 10.3389/fmed.2026.1806419

**Published:** 2026-04-07

**Authors:** Huiqing Guo

**Affiliations:** Outpatient Department of Pediatrics, Children's Hospital of Shanxi and Women Health Center of Shanxi, Taiyuan, China

**Keywords:** azithromycin, children, clinical efficacy, intravenous immunoglobulin, *Mycoplasma pneumoniae* pneumonia, sequential therapy

## Abstract

**Background:**

*Mycoplasma pneumoniae* pneumonia (MPP) is a common pediatric respiratory infection that can lead to serious complications. Increasing macrolide resistance and limited efficacy of antibiotic monotherapy necessitate exploration of adjunctive treatment strategies.

**Methods:**

This single-center retrospective, non-randomized cohort study included 140 children diagnosed with MPP between January 2020 and February 2022. Patients received either azithromycin sequential therapy alone (control group, *n* = 70) or IVIG plus azithromycin (IVIG group, *n* = 70). Baseline characteristics were comparable between groups; however, disease severity was not formally stratified. Outcomes included clinical efficacy, time to symptom resolution, inflammatory cytokines (IL-6, TNF, IL-2), immunoglobulin levels (IgG, IgA, IgM), pulmonary function, and adverse events. Multivariable logistic regression was used to adjust for baseline covariates.

**Results:**

The total effective rate was higher in the IVIG group than in the control group (97.14% vs. 84.29%, *p* < 0.05). Time to fever resolution, cough disappearance, wet rales resolution, and radiographic absorption was shorter in the IVIG group (all *p* < 0.01). IL-6 and TNF decreased and IL-2 increased in both groups, with greater changes in the IVIG group (*p* < 0.05). Post-treatment immunoglobulin levels and pulmonary function were higher in the IVIG group. In adjusted analysis, IVIG treatment was associated with a higher likelihood of clinical response (adjusted OR 5.82, 95% CI 1.22–27.74, *p* = 0.028). Adverse events were low and comparable between groups (*p* > 0.05).

**Conclusion:**

Adjunctive IVIG combined with azithromycin was associated with improved clinical and laboratory outcomes in children with MPP without increasing adverse events. Findings should be interpreted cautiously due to the retrospective design and potential residual confounding.

## 1 Introduction

*Mycoplasma pneumoniae* pneumonia (MPP) is a common cause of community-acquired pneumonia in children, accounting for approximately 10–20% of cases worldwide and representing a significant clinical burden in pediatric populations (1, 2). MPP exhibits a heterogeneous clinical spectrum, ranging from mild, self-limiting respiratory illness to severe or refractory disease characterized by persistent fever, progressive radiographic involvement, and extrapulmonary complications. In severe cases, MPP may involve multiple organ systems, including the cardiovascular, neurological, and hematological systems, and may lead to significant morbidity (3, 4). Because *Mycoplasma pneumoniae* lacks a cell wall, *β*-lactam antibiotics are ineffective, and macrolides remain the first-line treatment in children (5). However, the increasing prevalence of macrolide-resistant strains, particularly in East Asia, has emerged as a major challenge in clinical management, with resistance rates reported to exceed 80% in some regions (6). Although alternative antibiotics such as fluoroquinolones and tetracyclines are effective against resistant strains, their use in pediatric populations is limited due to safety concerns (7, 8). Consequently, treatment failure or delayed clinical recovery is increasingly recognized in a subset of patients.

Notably, refractory MPP is believed to be driven not only by antimicrobial resistance but also by an exaggerated host immune response, resulting in persistent inflammation and tissue injury (9). In this context, immunomodulatory therapies such as corticosteroids and intravenous immunoglobulin (IVIG) have been explored as adjunctive treatments. IVIG is not routinely recommended for all patients with MPP but is typically considered in selected severe or refractory cases in clinical practice (10). Its proposed mechanisms include modulation of inflammatory cytokine production, neutralization of pathogenic antibodies, and attenuation of immune-mediated tissue damage (11).

Although several studies have suggested potential benefits of IVIG in pediatric MPP, the current evidence remains limited and heterogeneous, with variability in study design, patient selection, and outcome definitions, leading to inconsistent conclusions (12, 13). Furthermore, data regarding the effects of IVIG on inflammatory cytokines, immune function, and pulmonary function in children with MPP are still insufficient. Therefore, this retrospective cohort study aimed to evaluate the association between adjunctive IVIG therapy and clinical outcomes in children with MPP, including symptom resolution, inflammatory cytokine profiles, immunoglobulin levels, pulmonary function, and short-term safety.

## 2 Materials and methods

### 2.1 Ethics statement

This study was approved by the Ethics Committee of Children’s Hospital of Shanxi and Women Health Center of Shanxi, Taiyuan 030013, China (Approval number ET-2023-0128A). As this was a retrospective study involving anonymized clinical data of pediatric patients, the requirement for informed consent from patients or their legal guardians was waived by the Ethics Committee in accordance with national regulations and the Declaration of Helsinki.

### 2.2 Study design

**Figure 1:**
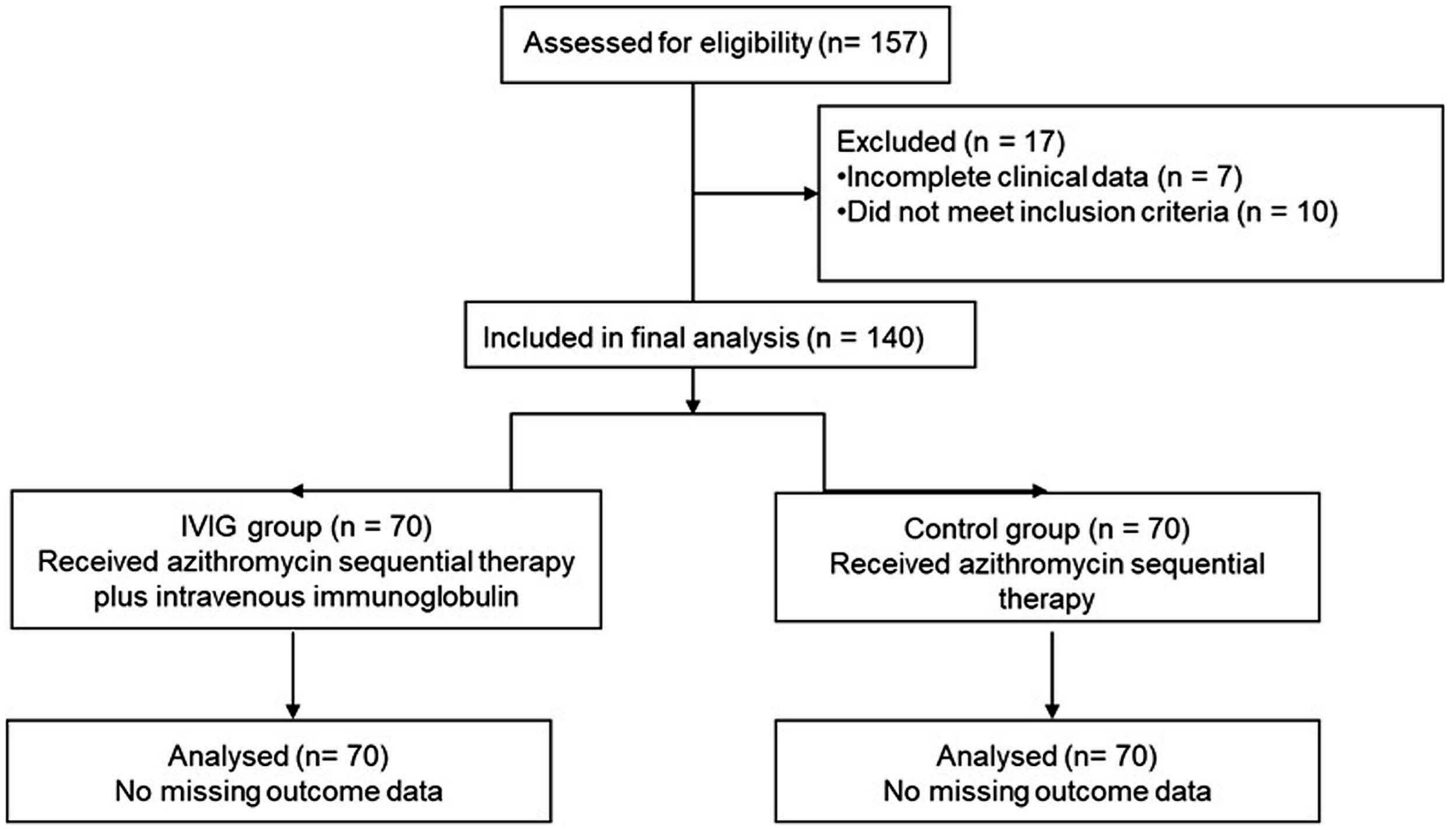
Study flowchart. Flow diagram illustrating the retrospective patient selection process, including initial screening of pediatric patients diagnosed with *Mycoplasma pneumoniae* pneumonia, application of inclusion and exclusion criteria, reasons for exclusion, and final classification into the IVIG group (*n* = 70) and the control group (*n* = 70) for analysis.

This was a single-center retrospective, non-randomized cohort study. A total of 157 pediatric patients diagnosed with *Mycoplasma pneumoniae* pneumonia were initially screened between January 2020 and February 2022 at Children’s Hospital of Shanxi and Women Health Center of Shanxi, Taiyuan 030013, China. Seventeen patients were excluded due to incomplete clinical data or failure to meet the inclusion criteria, including 10 patients from the IVIG group and 7 patients from the control group. No formal analysis of exclusion bias was performed due to the retrospective nature of the study. Ultimately, 140 eligible patients were included in the final analysis, comprising 70 patients in the IVIG group and 70 patients in the control group. No *a priori* sample size calculation or protocol registration was performed due to the retrospective nature of the study. The patient selection process is illustrated in Figure 1.

### 2.3 Inclusion and exclusion criteria

Children were eligible for inclusion if they were diagnosed with *Mycoplasma pneumoniae* pneumonia based on compatible clinical manifestations, radiographic findings, and serological evidence, in accordance with published diagnostic criteria (14). Eligible patients typically presented with a normal or mildly elevated peripheral white blood cell count accompanied by an increased erythrocyte sedimentation rate and showed a poor clinical response to *β*-lactam antibiotics, including penicillins or cephalosporins. Serological confirmation was defined as a serum cold agglutinin titer ≥1:32 and/or a *Mycoplasma pneumoniae*–specific antibody titer ≥1:80. These criteria reflect routine clinical practice at our institution; however, microbiological confirmation by PCR was not consistently available, which may limit diagnostic specificity. Only patients with complete clinical, laboratory, and imaging data available for analysis were included.

Patients were excluded if they had severe underlying diseases involving the cardiovascular, neurological, hepatic, renal, or hematopoietic systems, or if they presented with severe infection complicated by respiratory failure. Additional exclusion criteria included a known allergy to azithromycin or immunoglobulin preparations, respiratory symptoms attributable to alternative diagnoses (such as tuberculosis, chronic sinusitis, rhinitis, or airway foreign body), and incomplete medical records that precluded reliable assessment of clinical outcomes. No stratification by disease severity (e.g., mild vs. refractory MPP) was performed due to limitations in retrospective data availability.

### 2.4 Treatment regimens

All patients received routine supportive treatment after admission, including airway management, adequate fluid and nutritional support, and expectorant therapy when clinically indicated. Patients in the control group received azithromycin sequential therapy, consisting of intravenous azithromycin at a dose of 10 mg/kg once daily for 5 days, followed by a 4-day drug-free interval and subsequent oral azithromycin suspension at 10 mg/kg once daily for 3 days.

In addition to azithromycin sequential therapy, patients in the IVIG group received intravenous immunoglobulin (IVIG) as adjunctive immunomodulatory treatment. The IVIG regimen consisted of 1 g/kg/day administered intravenously once daily for 2 consecutive days, generally initiated within the first 48–72 h after admission based on clinical assessment; however, exact timing varied across patients. All patients in the IVIG group received the same dose and duration of IVIG. Initiation of IVIG was determined by the treating physician based on clinical judgment, which introduces a potential risk of confounding by indication inherent to retrospective observational studies. Both treatment regimens were administered over an overall treatment period of approximately 2 weeks.

### 2.5 Outcome measures

#### 2.5.1 Clinical efficacy

Clinical efficacy was evaluated 2 weeks after treatment initiation. Patients were classified as cured if body temperature normalized and remained stable for at least three consecutive days, cough completely resolved, and chest radiography demonstrated complete absorption of pulmonary inflammatory lesions. Improvement was defined as normalization of body temperature for at least three consecutive days, marked relief or resolution of cough, and partial absorption of pulmonary lesions on chest imaging. Treatment failure was defined as persistent fever or cough with no radiographic improvement or evidence of lesion progression. For statistical analysis, clinical response was defined as cured or improved versus ineffective. The total effective rate was calculated as the proportion of cured and improved cases relative to the total number of patients. This composite outcome reflects clinical practice but may be subject to subjectivity and variability in assessment.

#### 2.5.2 Time to symptom resolution

Time to symptom resolution was retrospectively obtained from medical records. The duration to fever resolution, disappearance of cough, disappearance of wet rales on auscultation, and radiographic absorption of pulmonary lesions were recorded based on daily clinical documentation and imaging reports. These time intervals were compared between the two treatment groups.

#### 2.5.3 Cytokine measurement

Serum inflammatory cytokine levels, including interleukin-6 (IL-6), tumor necrosis factor (TNF), and interleukin-2 (IL-2), were retrospectively analyzed. Venous blood samples had been routinely collected at hospital admission (before treatment) and approximately 2 weeks after treatment initiation as part of standard clinical care. Serum samples were stored at −70 °C and cytokine concentrations were measured using enzyme-linked immunosorbent assay (ELISA) according to the manufacturer’s instructions.

#### 2.5.4 Pulmonary function testing

Pulmonary function data were retrospectively obtained from patients’ medical records. Pulmonary function tests were performed at baseline (before treatment) and 2 weeks after treatment initiation using a MasterScreen spirometer (CareFusion, Germany), in accordance with standard testing procedures. The assessed parameters included forced vital capacity (FVC), forced expiratory volume in 1 s (FEV_1_), and peak expiratory flow (PEF). For each patient, three technically acceptable maneuvers were recorded, and the highest value for each parameter was used for statistical analysis. Given the young age of the study population, variability in test performance and cooperation cannot be excluded.

#### 2.5.5 Immunoglobulin levels

Serum immunoglobulin levels, including immunoglobulin G (IgG), immunoglobulin A (IgA), and immunoglobulin M (IgM), were retrospectively collected from laboratory records. Measurements were performed at admission and 2 weeks after treatment initiation using ELISA kits in accordance with the manufacturer’s protocols as part of routine clinical testing.

#### 2.5.6 Adverse events

Adverse events occurring during treatment were retrospectively identified through review of nursing records and physician notes. Recorded adverse events included nausea or vomiting, diarrhea, and abdominal pain. The incidence of adverse events was compared between the control group and the IVIG group. Due to the low frequency of adverse events, statistical comparisons were interpreted cautiously.

### 2.6 Statistical analysis

Statistical analyses were performed using SPSS software (IBM Corp., Armonk, NY, USA). Continuous variables were tested for normality using the Shapiro–Wilk test. Data with a normal distribution are presented as mean ± standard deviation (SD), while categorical variables are expressed as counts and percentages. Comparisons of continuous variables between the two groups were performed using the independent-samples *t*-test for normally distributed data. Paired *t*-tests were used to compare pre- and post-treatment measurements within the same group. Categorical variables, including clinical efficacy and incidence of adverse events, were compared using the chi-square (*χ*^2^) test or Fisher’s exact test when appropriate. To reduce potential confounding, multivariable logistic regression analysis was performed to evaluate factors independently associated with clinical response, adjusting for baseline covariates including age, sex, disease duration, baseline IL-6, and FEV_1_. The total effective rate was calculated as the proportion of cured and improved cases relative to the total number of patients in each group. Time-to-symptom resolution and laboratory parameters were analyzed as continuous variables. All statistical tests were two-sided, and a *p* value < 0.05 was considered statistically significant. Given the multiple secondary comparisons performed, no formal adjustment for multiple testing (e.g., Bonferroni correction) was applied; therefore, the results should be interpreted as exploratory. Given the retrospective design, no propensity score matching or sensitivity analysis was performed. Missing data were excluded from analysis, and no imputation was performed due to the retrospective nature of the study.

## 3 Results

### 3.1 Baseline characteristics

A total of 140 pediatric patients with *Mycoplasma pneumoniae* pneumonia were included in the final analysis, comprising 70 patients in the control group and 70 patients in the IVIG group. Baseline demographic and clinical characteristics were similar between the two groups, with no statistically significant differences observed for the measured variables. The mean age was 5.41 ± 0.33 years in the control group and 5.44 ± 0.31 years in the IVIG group. The control group included 47 boys and 23 girls, while the IVIG group included 41 boys and 29 girls. The mean disease duration was 12.24 ± 2.83 days in the control group and 12.73 ± 2.54 days in the IVIG group. Mean body mass index was 17.11 ± 1.39 kg/m^2^ in the control group and 16.86 ± 1.35 kg/m^2^ in the IVIG group. Baseline levels of inflammatory cytokines and pulmonary function parameters were also comparable between groups (Table 1).

**Table 1:** Baseline characteristics of pediatric patients with *Mycoplasma pneumoniae* pneumonia.

Characteristic	Control group (*n* = 70)	IVIG group (*n* = 70)	Mean difference	*p* value
Age (years)	5.41 ± 0.33	5.44 ± 0.31	0.03	0.63
Male, *n* (%)	47 (67.1%)	41 (58.6%)	–	0.29
Disease duration before admission (days)	12.24 ± 2.83	12.73 ± 2.54	0.49	0.34
Body mass index (kg/m^2^)	17.11 ± 1.39	16.86 ± 1.35	−0.25	0.27
Baseline IL-6 (pg/mL)	18.38 ± 3.91	18.35 ± 3.35	−0.03	0.96
Baseline TNF (pg/mL)	22.5 ± 4.1	22.8 ± 4.5	0.3	0.68
Baseline IL-2 (pg/mL)	6.8 ± 1.2	6.9 ± 1.1	0.1	0.61
Baseline FVC (L)	1.20 ± 0.21	1.21 ± 0.22	0.01	0.79
Baseline FEV1 (L)	1.01 ± 0.12	1.05 ± 0.15	0.04	0.11

Data are presented as mean ± SD or *n* (%).

### 3.2 Comparison of clinical efficacy

**Figure 2:**
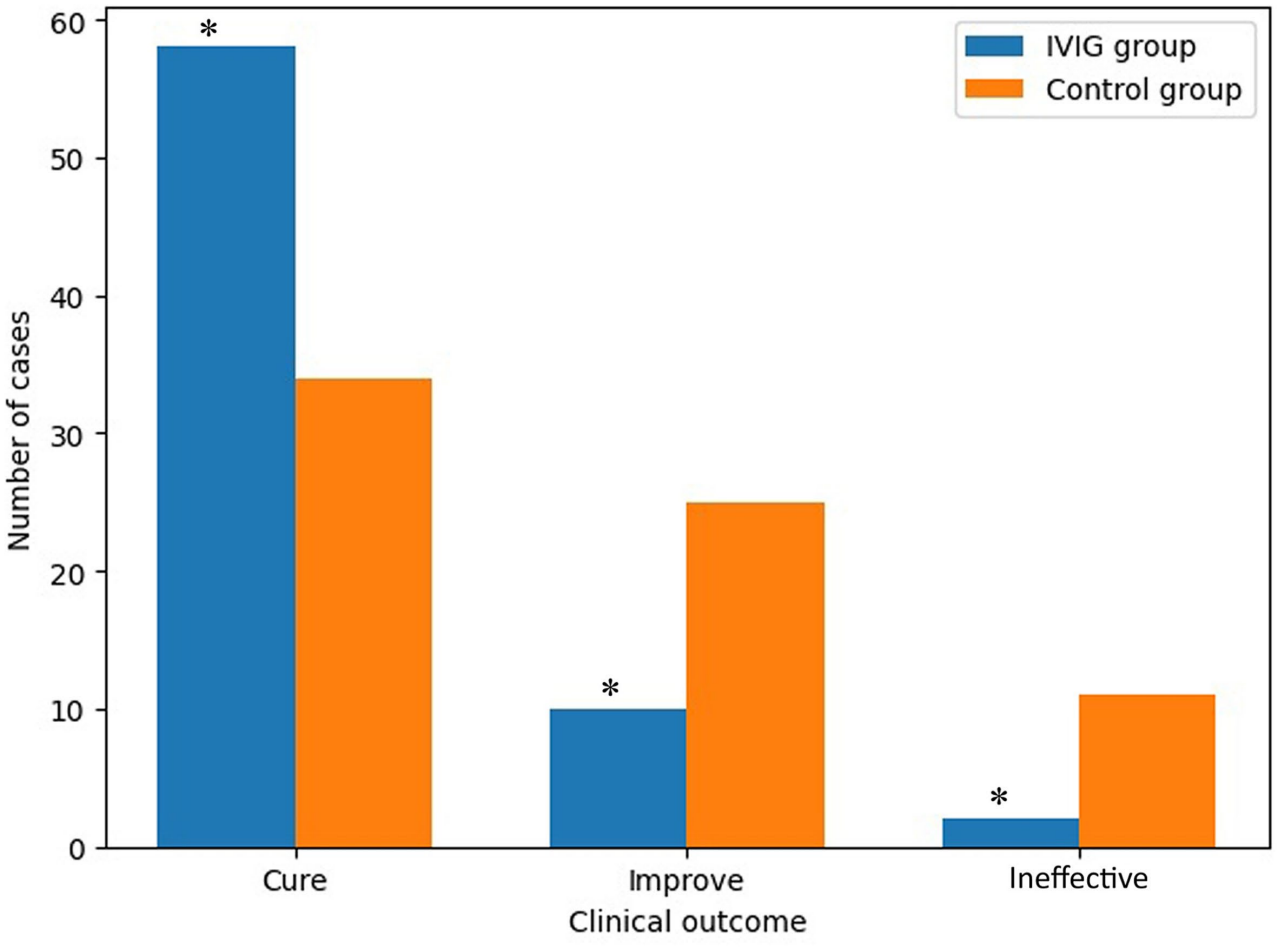
Comparison of clinical efficacy between groups. Bar chart showing the distribution of clinical outcomes (cure, improvement, and ineffective) in the IVIG and control groups. The IVIG group demonstrated a significantly higher cure rate and lower proportion of ineffective cases compared with the control group. *p* < 0.05 vs. control group (chi-square test).

Among patients in the IVIG group, 58 were classified as cured, 10 showed improvement, and 2 were classified as ineffective, yielding a total effective rate of 97.14%. In the control group, 34 patients were cured, 25 showed improvement, and 11 were classified as ineffective, resulting in a total effective rate of 84.29%. The total effective rate was higher in the IVIG group compared with the control group (*p* < 0.05). The comparison of clinical efficacy between the two groups is shown in Figure 2. In multivariable logistic regression analysis adjusting for baseline covariates, IVIG treatment was independently associated with a higher likelihood of clinical response (adjusted OR 5.82, 95% CI 1.22–27.74, *p* = 0.028) (Table 2).

**Table 2:** Multivariable logistic regression analysis of factors associated with clinical response.

Variable	Adjusted OR	95% CI	*p* value
IVIG treatment	5.82	1.22–27.74	0.028
Age	1.03	0.88–1.21	0.72
Male sex	0.91	0.32–2.59	0.86
Disease duration before admission	0.95	0.81–1.11	0.51
Baseline IL-6	0.96	0.89–1.03	0.25
Baseline FEV1	1.44	0.82–2.52	0.20

Outcome variable: Clinical response (cured or improved vs ineffective). Adjusted for age, sex, disease duration, baseline IL-6, and FEV1.

### 3.3 Time to resolution of clinical signs and symptoms

The time to resolution of major clinical symptoms was shorter in the IVIG group compared with the control group. Specifically, the duration to fever resolution, disappearance of cough, disappearance of wet rales, and radiographic absorption of pulmonary infiltrates were all reduced in patients receiving IVIG combined with azithromycin sequential therapy (all *p* < 0.01). The magnitude of these differences and corresponding 95% confidence intervals are presented in Table 3.

**Table 3:** Time to resolution of clinical symptoms.

Outcome (days)	Control group (*n* = 70)	IVIG group (*n* = 70)	Mean difference (IVIG—Control)	95% CI	*p* value
Fever resolution	4.59 ± 1.89	2.47 ± 0.31	−2.12	−2.75 to −1.49	<0.01^*^
Cough resolution	8.84 ± 2.21	5.59 ± 1.84	−3.25	−4.02 to −2.48	<0.01^*^
Wet rales resolution	9.83 ± 2.21	6.49 ± 2.93	−3.34	−4.29 to −2.39	<0.01^*^
Radiographic absorption	15.59 ± 2.28	12.29 ± 5.91	−3.30	−4.80 to −1.80	<0.01^*^

Data are presented as mean ± SD. ^*^p < 0.05 was considered statistically significant.

### 3.4 Changes in inflammatory cytokine levels

There were no significant differences in baseline serum levels of IL-6, TNF, or IL-2 between the two groups prior to treatment. After 2 weeks of treatment, serum IL-6 and TNF levels decreased in both groups, while IL-2 levels increased compared with baseline values (*p* < 0.05 within groups). Compared with the control group, the IVIG group showed lower post-treatment IL-6 and TNF levels and higher IL-2 levels (all *p* < 0.01). However, these findings should be interpreted cautiously, as no adjustment for potential confounding factors influencing inflammatory responses was performed. The detailed cytokine data are shown in Table 4.

**Table 4:** Changes in serum inflammatory cytokine levels.

Cytokine	Group	Before treatment	After treatment	Mean change	*p* value
IL-6 (pg/mL)	Control	18.38 ± 3.91	12.84 ± 2.92	−5.54	<0.05^*^
	IVIG	18.35 ± 3.35	8.38 ± 1.54	−9.97	<0.01^*^
TNF (pg/mL)	Control	22.5 ± 4.1	15.8 ± 3.9	−6.7	<0.05^*^
	IVIG	22.8 ± 4.5	9.6 ± 2.7	−13.2	<0.01^*^
IL-2 (pg/mL)	Control	6.8 ± 1.2	10.9 ± 2.8	+4.1	<0.05^*^
	IVIG	6.9 ± 1.1	14.6 ± 3.1	+7.7	<0.01^*^

Data are presented as mean ± SD. ^*^p < 0.05 was considered statistically significant.

### 3.5 Changes in immunoglobulin levels

Baseline serum levels of IgG, IgA, and IgM were comparable between the two groups. After treatment, levels of all three immunoglobulins increased in both groups compared with baseline values (*p* < 0.05 within groups). Post-treatment IgG, IgA, and IgM levels were higher in the IVIG group than in the control group (all *p* < 0.01). Detailed immunoglobulin levels before and after treatment are summarized in Table 5.

**Table 5:** Changes in serum immunoglobulin levels.

Parameter	Group	Before	After	Mean change	*p* value
IgG (g/L)	Control	5.03 ± 0.41	6.12 ± 0.75	+1.09	<0.05^*^
	IVIG	5.05 ± 0.23	9.48 ± 1.23	+4.43	<0.01^*^
IgA (g/L)	Control	0.69 ± 0.11	1.28 ± 0.22	+0.59	<0.05^*^
	IVIG	0.70 ± 0.17	1.48 ± 0.32	+0.78	<0.01^*^
IgM (g/L)	Control	1.05 ± 0.31	1.25 ± 0.23	+0.20	<0.05^*^
	IVIG	1.07 ± 0.35	1.79 ± 0.21	+0.72	<0.01^*^

Data are presented as mean ± SD. ^*^p < 0.05 was considered statistically significant.

### 3.6 Comparison of pulmonary function

Pulmonary function parameters, including FVC, FEV_1_, and PEF, did not differ significantly between the two groups before treatment. After 2 weeks of treatment, pulmonary function indices improved in both groups. Post-treatment FVC, FEV_1_, and PEF values were higher in the IVIG group compared with the control group (all *p* < 0.01). The between-group differences with corresponding 95% confidence intervals are presented in Table 6.

**Table 6:** Changes in pulmonary function parameters.

Parameter	Control group (*n* = 70)	IVIG group (*n* = 70)	Mean difference (IVIG—Control)	95% CI	*p* value
FVC after treatment (L)	1.25 ± 0.14	1.38 ± 0.12	0.13	0.07–0.19	<0.01^*^
FEV1 after treatment (L)	1.15 ± 0.15	1.25 ± 0.11	0.10	0.04–0.16	<0.01^*^
PEF after treatment (L/s)	2.60 ± 0.27	2.99 ± 0.21	0.39	0.28–0.50	<0.01^*^

Data are presented as mean ± SD. ^*^p < 0.05 was considered statistically significant.

### 3.7 Incidence of adverse events

During treatment, adverse events were mild and infrequent in both groups. In the control group, two patients experienced nausea or vomiting and one patient developed diarrhea. In the IVIG group, two patients experienced nausea or vomiting and one patient reported abdominal pain. No statistically significant difference in the incidence of adverse events was observed between the two groups (*p* > 0.05).

**Figure 3:**
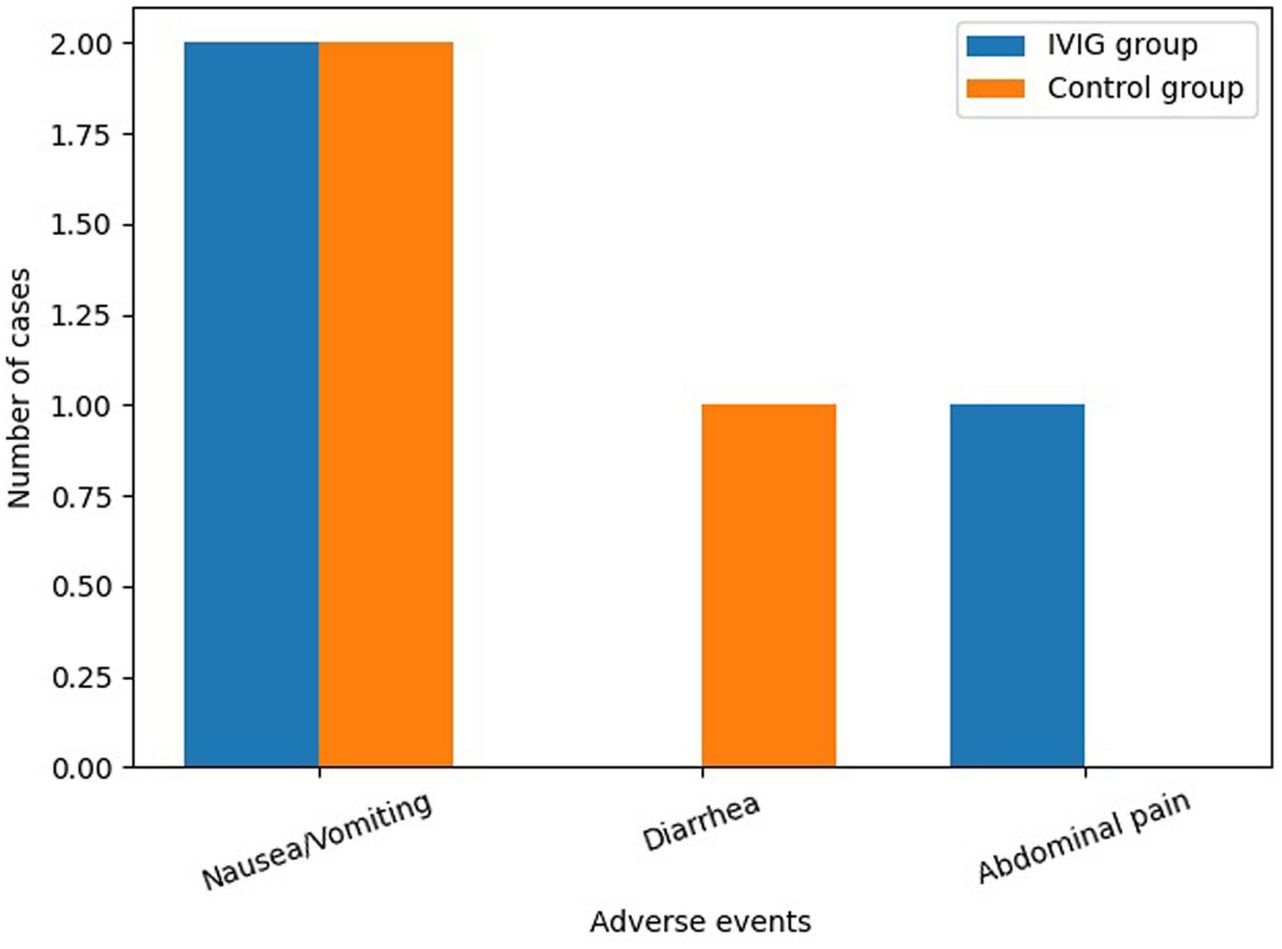
Incidence of adverse events in both groups. Bar chart illustrating the distribution of adverse events, including nausea/vomiting, diarrhea, and abdominal pain, in the IVIG and control groups. Adverse events were infrequent and mild in both groups, with no statistically significant differences between groups.

Given the low frequency of adverse events, these comparisons should be interpreted with caution. The distribution of adverse events is shown in Figure 3.

## 4 Discussion

*Mycoplasma pneumoniae* (MP) is a common pathogen responsible for respiratory tract infections in children and adolescents, ranging from mild upper respiratory tract involvement to severe and potentially fatal pneumonia. *Mycoplasma pneumoniae* pneumonia (MPP) is one of the leading causes of community-acquired pneumonia (CAP) in pediatric populations, accounting for approximately 10–20% of cases (2, 3, 15). MP infection is widely distributed geographically and is primarily transmitted via respiratory droplets, with high infectivity (16, 17). Seasonal variation has been observed, with higher incidence in autumn in northern China and winter in southern regions (18). Because MP lacks a cell wall, *β*-lactam antibiotics such as penicillins and cephalosporins are ineffective, making macrolides the cornerstone of treatment (19, 20). However, the increasing prevalence of macrolide-resistant MP strains—particularly in East Asia—has posed significant challenges to clinical management, with resistance rates in China reported to range from 63 to 88% (21). Consequently, there is growing interest in adjunctive therapeutic strategies for selected patients with more severe or refractory disease.

Current management of MPP relies on symptomatic supportive care combined with antimicrobial therapy. Macrolide antibiotics inhibit protein synthesis in MP by targeting the bacterial ribosome and remain the first-line treatment for pediatric patients (22). Azithromycin, a second-generation macrolide, is widely used due to its favorable pharmacokinetic profile, including high tissue penetration, prolonged half-life, and sustained antimicrobial activity after discontinuation (23, 24). Compared with erythromycin, azithromycin is associated with fewer gastrointestinal adverse effects and reduced hepatotoxicity, making it more suitable for children (25). Nevertheless, azithromycin monotherapy may be insufficient in some cases, particularly in the context of macrolide resistance or more severe inflammatory responses. Therefore, combining azithromycin with immunomodulatory therapies has been explored as a potential approach to improve clinical outcomes in pediatric MPP. Immunoglobulins play a critical role in humoral immunity and host defense. Immunoglobulin molecules consist of two identical heavy chains and two identical light chains, enabling specific antigen recognition and immune regulation (26–37). Immunoglobulin G (IgG), the predominant immunoglobulin in serum, provides passive immunity and enhances pathogen neutralization, complement activation, and immune modulation (27). Secretory immunoglobulins contribute to mucosal immunity by inhibiting microbial adhesion to respiratory epithelial surfaces (28, 38–40). Intravenous immunoglobulin (IVIG) therapy has been applied in selected cases of refractory MPP due to its immunomodulatory properties, including modulation of inflammatory responses and attenuation of immune-mediated tissue injury (29, 30, 41–44). However, its routine use remains controversial and is not universally recommended in clinical guidelines.

In the present retrospective study, patients receiving IVIG combined with azithromycin sequential therapy were observed to have higher clinical response rates and shorter symptom resolution times compared with those receiving azithromycin alone. After adjustment for selected baseline variables, IVIG treatment remained associated with an increased likelihood of clinical response. In addition, reductions in IL-6 and TNF levels and increases in IL-2 levels were more pronounced in the IVIG group. Post-treatment immunoglobulin levels and pulmonary function parameters were also higher in the IVIG group. These findings suggest a potential association between adjunctive IVIG therapy and improved clinical and laboratory outcomes; however, causal inference cannot be established due to the observational study design.

The observed differences in inflammatory markers and immunoglobulin levels should be interpreted with caution, as these parameters may be influenced by disease severity, treatment timing, and other unmeasured confounders. Similarly, improvements in pulmonary function may reflect overall clinical recovery rather than a direct effect of IVIG. Given that IVIG administration was based on physician discretion, confounding by indication remains a key limitation and may have influenced the observed outcomes. Importantly, the incidence of adverse events was low and comparable between groups, suggesting that IVIG was generally well tolerated in this cohort. However, the small number of events limits the ability to draw definitive conclusions regarding safety. In addition, the cost and resource implications of IVIG should be considered in clinical decision-making. IVIG is an expensive therapy and its routine use in all patients with MPP may not be justified, particularly in resource-limited settings. Therefore, its use should be reserved for carefully selected patients with severe or refractory disease, in accordance with clinical judgment and existing evidence (3, 10).

Several limitations of this study should be acknowledged. First, this was a single-center retrospective cohort analysis, which may have introduced selection bias and limits the generalizability of the findings. Second, treatment allocation was based on clinical judgment rather than randomization, and residual confounding from unmeasured variables cannot be excluded. In addition, multiple comparisons were conducted without formal adjustment, which may increase the risk of type I error. Third, no standardized criteria for defining disease severity or refractory MPP were applied, which may affect the interpretation of treatment indications. Fourth, the diagnosis of MPP was based primarily on serological criteria without consistent molecular confirmation, which may have affected diagnostic accuracy. Moreover, in addition, pulmonary function testing in young children (mean age ~5 years) may be limited by suboptimal cooperation and measurement variability, which could affect the reliability of these results. Fifth, the sample size was relatively modest, and long-term follow-up data were unavailable, preventing evaluation of disease recurrence and long-term pulmonary outcomes. In addition, detailed information regarding macrolide resistance genotypes of *Mycoplasma pneumoniae* was not available, which may have provided further insight into treatment responsiveness.

Therefore, prospective, multicenter randomized controlled studies with larger sample sizes and longer follow-up periods are warranted to further evaluate the clinical role, optimal indications, and safety profile of intravenous immunoglobulin as an adjunctive therapy in pediatric MPP.

## 5 Conclusion

In conclusion, the findings of this retrospective study indicate that adjunctive intravenous immunoglobulin combined with azithromycin sequential therapy was associated with improved clinical response, more favorable inflammatory profiles, and enhanced immune-related parameters in children with *Mycoplasma pneumoniae* pneumonia. However, these associations should be interpreted with caution, as causal relationships cannot be established due to the non-randomized study design and potential confounding factors. This treatment strategy appeared to be well tolerated, with no increase in the incidence of adverse events observed in this cohort. Given the methodological limitations, including retrospective design, potential confounding by indication, and relatively limited sample size, these findings should be considered exploratory. Prospective multicenter studies with larger sample sizes and standardized treatment indications are warranted to further clarify the clinical role and safety of intravenous immunoglobulin in pediatric MPP.

## Data Availability

The original contributions presented in the study are included in the article/supplementary material, further inquiries can be directed to the corresponding author.

## References

[1] Subspecialty Group of Respiratory, the Society of Pediatrics, Chinese Medical Association, China National Clinical Research Center of Respiratory Diseases, Editorial Board, Chinese Journal of Pediatrics. Evidence-based guideline for the diagnosis and treatment of *Mycoplasma pneumoniae* pneumonia in children (2023). Pediatr Investig. (2025) 9:1. doi: 10.1002/ped4.12469 PMC11998179 40241891

[2] Tsai TA Tsai CK Kuo KC Yu HR. Rational stepwise approach for *Mycoplasma pneumoniae* pneumonia in children. J Microbiol Immunol Infect. (2021) 54:557–65. doi: 10.1016/j.jmii.2020.10.002, 33268306

[3] Tong L Huang S Zheng C Zhang Y Chen Z. Refractory *Mycoplasma pneumoniae* pneumonia in children: early recognition and management. J Clin Med. (2022) 11:2824. doi: 10.3390/jcm11102824, 35628949 PMC9144103

[4] Kutty PK Jain S Taylor TH Bramley AM Diaz MH Ampofo K . *Mycoplasma pneumoniae* among children hospitalized with community-acquired pneumonia. Clin Infect Dis. (2019) 68:5–12. doi: 10.1093/cid/ciy419, 29788037 PMC6552676

[5] Bradley JS Byington CL Shah SS Alverson B Carter ER Harrison C . The management of community-acquired pneumonia in infants and children older than 3 months of age: clinical practice guidelines by the Pediatric Infectious Diseases Society and the Infectious Diseases Society of America. Clin Infect Dis. (2011) 53:e25–76. doi: 10.1093/cid/cir531, 21880587 PMC7107838

[6] Wang YS Zhou YL Bai GN Li SX Xu D Chen LN . Expert consensus on the diagnosis and treatment of macrolide-resistant *Mycoplasma pneumoniae* pneumonia in children. World J Pediatr. (2024) 20:901–14. doi: 10.1007/s12519-024-00831-0, 39143259 PMC11422262

[7] Jang MS Kim BG Kim J. Prediction model for prolonged fever in patients with *Mycoplasma pneumoniae* pneumonia: a retrospective study of 716 pediatric patients. BMC Pulm Med. (2021) 21:168. doi: 10.1186/s12890-021-01534-2, 34006256 PMC8130327

[8] Shah SS. *Mycoplasma pneumoniae* as a cause of community-acquired pneumonia in children. Clin Infect Dis. (2019) 68:13–4. doi: 10.1093/cid/ciy421, 29788200

[9] Liu F Wang Q Cheng Q Zhang H. Immune activation and mucin dysregulation in pediatric refractory *Mycoplasma pneumoniae* pneumonia with mucus plugs. Front Cell Infect Microbiol. (2025) 15:1706340. doi: 10.3389/fcimb.2025.1706340, 41561092 PMC12812597

[10] Sharplin L Goyal V. *Mycoplasma pneumoniae* respiratory tract infections in children: when and how to diagnose and treat. Breathe. (2025) 21:250046. doi: 10.1183/20734735.0046-2025, 41098325 PMC12519950

[11] Yang EA Lee KY. Are alternative antibiotics needed for antibiotic-nonresponsive *Mycoplasma pneumoniae* pneumonia? Clin Exp Pediatr. (2020) 63:44–5. doi: 10.3345/kjp.2019.00332, 32066227 PMC7029668

[12] Marino S Pavone P Marino L Nunnari G Ceccarelli M Coppola C . SARS-CoV-2: the impact of co-infections with particular reference to *Mycoplasma pneumoniae*. Microorganisms. (2022) 10:1936. doi: 10.3390/microorganisms10101936 36296214 PMC9610609

[13] Kim YS Lee YY Lee E. Cases of macrolide-resistant *Mycoplasma pneumoniae* pneumonia-associated pulmonary thromboembolism. Pediatr Pulmonol. (2021) 56:1796–9. doi: 10.1002/ppul.25298 33559952

[14] Rogozinski LE Alverson BK Biondi EA. Diagnosis and treatment of *Mycoplasma pneumoniae* in children. Minerva Pediatr. (2017) 69:156–60. doi: 10.23736/S0026-4946.16.04866-0, 28178776

[15] Gordon O Oster Y Michael-Gayego A Marans RS Averbuch D Engelhard D . The clinical presentation of pediatric *Mycoplasma pneumoniae* infections. Pediatr Infect Dis J. (2019) 38:698–705. doi: 10.1097/INF.0000000000002291 30985519

[16] Davis J Ericson JE Kavanagh R. Severe pediatric *Mycoplasma pneumoniae* infection requiring extracorporeal membrane oxygenation. Pediatr Infect Dis J. (2021) 40:154–6. doi: 10.1097/INF.0000000000003051 33427801

[17] Kumar S Bharti PK Baveja CP Mantan M Saigal SR Garg IB. Detection of *Mycoplasma pneumoniae* by PCR in pediatric lower respiratory tract infections. Indian J Med Microbiol. (2022) 40:250–3. doi: 10.1016/j.ijmmb.2022.01.002, 35063301

[18] Salma Fahmidha M Karunanandhan M Munigangaiah L Srinivasaraghavan R. *Mycoplasma pneumoniae*-associated mucositis. Indian J Pediatr. (2021) 88:183. doi: 10.1007/s12098-020-03417-6 32591998

[19] Meyer Sauteur PM Trück J van Rossum AMC Berger C. Circulating antibody-secreting cell response during *Mycoplasma pneumoniae* pneumonia. J Infect Dis. (2020) 222:136–47. doi: 10.1093/infdis/jiaa062, 32034406

[20] De Luigi G Meoli M Zgraggen L Kottanattu L Simonetti GD Terrani I . Mucosal respiratory syndrome: a systematic review. Dermatology. (2022) 238:53–9. doi: 10.1159/000514815, 33774629 PMC8089407

[21] Copete AR Vera C Herrera M Aguilar YA Rueda ZV Vélez LA. *Mycoplasma pneumoniae* in children with and without pneumonia. Pediatr Infect Dis J. (2020) 39:104–8. doi: 10.1097/INF.0000000000002636 32118860

[22] Choo S Kim SH Lee E. Clinical significance of *Mycoplasma pneumoniae*-specific IgM titers. BMC Infect Dis. (2022) 22:470. doi: 10.1186/s12879-022-07456-6 35578177 PMC9109195

[23] Piras C Pintus R Pruna D Dessì A Atzori L Fanos V. Pediatric acute-onset neuropsychiatric syndrome associated with *Mycoplasma pneumoniae*. Curr Pediatr Rev. (2020) 16:183–93. doi: 10.2174/1573396315666191022102925, 31642785 PMC8193809

[24] Zhang XB He W Gui YH Lu Q Yin Y Zhang JH . Current *Mycoplasma pneumoniae* epidemic among children in Shanghai: unusual pneumonia caused by usual pathogen. World J Pediatr. (2024) 20:5–10. doi: 10.1007/s12519-023-00793-9, 38231466

[25] Shim JY. Current perspectives on atypical pneumonia in children. Clin Exp Pediatr. (2020) 63:469–76. doi: 10.3345/cep.2019.00360, 32517424 PMC7738772

[26] Valoti E Piras R Mele C Alberti M Liguori L Breno M . *Mycoplasma pneumoniae* infection associated with anti-factor H autoantibodies. Nephron. (2022) 146:593–8. doi: 10.1159/000523998 35405682

[27] Esposito S Argentiero A Gramegna A Principi N. *Mycoplasma pneumoniae*: unresolved therapeutic challenges. Expert Opin Pharmacother. (2021) 22:1193–202. doi: 10.1080/14656566.2021.1882420, 33544008

[28] Thangaraju S Bagri N Gupta V Sinha A. Mycoplasma-induced rash and mucositis. Indian J Pediatr. (2021) 88:802–4. doi: 10.1007/s12098-021-03658-z 33447929

[29] Kim HS Sol IS Li D Choi M Choi YJ Lee KS . Glucocorticoids for macrolide-refractory *Mycoplasma pneumoniae*. BMC Pulm Med. (2019) 19:251. doi: 10.1186/s12890-019-0990-8 31852460 PMC6921474

[30] Roshan S Tan SW. Severe *Mycoplasma pneumoniae* with autoimmune hemolytic anemia. Med J Malaysia. (2020) 75:600–2. Available online at: https://www.e-mjm.org/2020/v75n5/mycoplasma-pneumonia.pdf 32918437

[31] Lee E Choi I. Clinical usefulness of serum lactate dehydrogenase levels in *Mycoplasma pneumoniae* pneumonia in children. Indian J Pediatr. (2022) 89:1003–9. doi: 10.1007/s12098-022-04205-0, 35665905

[32] Cho HK. Consideration in treatment decisions for refractory *Mycoplasma pneumoniae* pneumonia. Clin Exp Pediatr. (2021) 64:459–67. doi: 10.3345/cep.2020.01305, 33561337 PMC8426095

[33] Kim SH Lee E Song ES Lee YY. Clinical significance of pleural effusion in *Mycoplasma pneumoniae* pneumonia in children. Pathogens. (2021) 10:1075. doi: 10.3390/pathogens10091075, 34578108 PMC8469935

[34] Choo S Lee YY Lee E. Clinical significance of respiratory virus coinfection in children with *Mycoplasma pneumoniae* pneumonia. BMC Pulm Med. (2022) 22:212. doi: 10.1186/s12890-022-02005-y, 35637540 PMC9150047

[35] Ramien ML Bruckner AL. Mucocutaneous eruptions in acutely ill pediatric patients—think of *Mycoplasma pneumoniae*. JAMA Dermatol. (2020) 156:124–5. doi: 10.1001/jamadermatol.2019.3589, 31851301

[36] Graf G Vassalli GAM Kottanattu L Bianchetti MG Agostoni C Milani GP . Acute pancreatitis associated with atypical bacterial pneumonia: a systematic review. J Clin Med. (2022) 11:7248. doi: 10.3390/jcm11237248 36498822 PMC9736890

[37] Varghese SM Kerkar VV. Macrolide resistant *Mycoplasma pneumoniae* in community-acquired pneumonia. Indian J Pediatr. (2020) 87:958. doi: 10.1007/s12098-020-03301-3, 32333367

[38] Roh EJ Shim JY Chung EH. Epidemiology and surveillance of community-acquired pneumonia in children. Clin Exp Pediatr. (2022) 65:563–73. doi: 10.3345/cep.2022.00374, 36265520 PMC9742763

[39] Okumura T Kawada JI Tanaka M Narita K Ishiguro T Hirayama Y . Corticosteroid therapy for refractory *Mycoplasma pneumoniae* pneumonia. J Infect Chemother. (2019) 25:346–50. doi: 10.1016/j.jiac.2019.01.003 30718192

[40] Wrotek A Robakiewicz J Pawlik K Rudzinski P Pilarska I Jaroń A . Etiology of pneumonia correlates with inflammatory markers. J Clin Med. (2022) 11:5506. doi: 10.3390/jcm11195506 36233374 PMC9571658

[41] Oishi T Yoshioka D Nakano T Ouchi K. Antimicrobial susceptibility of *Mycoplasma pneumoniae*. Microorganisms. (2022) 10:2428. doi: 10.3390/microorganisms10122428 36557681 PMC9787913

[42] Meyer Sauteur PM Krautter S Ambroggio L Seiler M Paioni P Relly C . Biomarkers predicting *Mycoplasma pneumoniae* pneumonia. Clin Infect Dis. (2020) 71:1645–54. doi: 10.1093/cid/ciz1059 31665253 PMC7108170

[43] Vinodhini P Peterson RR Hanmantgad S Lakshmi KS. Mycoplasma-associated pediatric stroke. J Fam Med Prim Care. (2022) 11:3346–8. doi: 10.4103/jfmpc.jfmpc_2056_21, 36119273 PMC9480644

[44] Choi JH Seong GM Ko Y Kim YR Kim C. Macrolide-resistant *Mycoplasma pneumoniae* pneumonia in adults. Microb Drug Resist. (2019) 25:577–81. doi: 10.1089/mdr.2018.0295, 30484744

